# Analysing Siamese Neural Network Architectures for Computing Name Similarity.

**DOI:** 10.23889/ijpds.v7i3.2077

**Published:** 2022-08-25

**Authors:** Nicholas Vinden, Jeremy Foxcroft, Luiza Antonie

**Affiliations:** 1 University of Guelph

## Introduction

A wide assortment of string similarity measures can be used to determine how similar two names are. A diverse set of discriminating and independent features for name similarity are important for classification during record linkage. A Siamese neural network could surpass traditional string similarity measures for the name similarity problem.

## Approach

This research aims to compare a classifier based on the Siamese network architecture with a Random Forest classifier. In addition to comparing overall performance, we seek to answer whether there are any special properties of certain matching name pairs where the complexity of the Siamese network offers particular benefit.

Our data consists of 25,000 last name pairings, with each pair being two variants of a family name. Name similarity predictions from the Siamese network are compared to a Random Forest model that serves as an ensemble of existing string similarity measures.

## Results

We compare the similarity scores yielded by the two methods and discuss the results. We describe the representation of names to each method; name representation is computed formulaically for the traditional measures but is learned by the Siamese network during training. The comparison of different methods is made both in terms of their similarity prediction quality, and the computational cost to generate the predictions.

As expected, the Siamese network necessitates a significant computational cost to train. Unexpectedly, the ensemble of traditional measures yields almost identical overall classification performance. However, we expect that further analysis of false positives and false negatives will yield some insight into when practitioners should consider one method over the other.

## Conclusions/Implications

Results suggest that there may be instances where a Siamese network outperforms other similarity measures, although training a Siamese network comes at a considerable computational cost. It is worth considering this approach to name similarity as an additional similarity feature when performing record linkage tasks.

